# Tenascin X and Tenascin C Serum Levels in Relation to Age and Cardiometabolic Risk Parameters

**DOI:** 10.3390/ijms27209180

**Published:** 2026-10-16

**Authors:** Sabina Baumgartner-Parzer, Konstantin Fuhrberg, Christine Wagenlechner, Franziska Schnait, Nina Schmid, Michaela Bayerle-Eder, Gabriele Grimm, Greisa Vila, Marie Helene Schernthaner-Reiter

**Affiliations:** 1 Clinical Division of Endocrinology and Metabolism, Department of Internal Medicine III, Medical University of Vienna, Waehringer Guertel 18-20, 1090 Vienna, Austria; 2 Institute for Medical Statistics, Medical University of Vienna, Spitalgasse 23, 1090 Vienna, Austria; 3 Department of Laboratory Medicine, Medical University of Vienna, Waehringer Guertel 18-20, 1090 Vienna, Austria

**Keywords:** Connective tissue diseases, Ehlers–Danlos syndrome, congenital adrenal hyperplasia, cardiometabolic risk factors

## Abstract

Tenascin C (TNC) and tenascin X (TNX) are extracellular matrix glycoproteins in the same family but with reportedly distinct functions: TNC is acutely up-regulated during tissue damage, whereas TNX maintains structural connective tissue stability. Recently, recurring pathogenic variants in the TNX gene (*TNXB*) were linked to Ehlers–Danlos syndrome in patients with congenital adrenal hyperplasia, yet the regulation and clinical function of TNX remain poorly understood. In this retrospective cohort study, serum TNX and TNC were measured by ELISA in 274 patients with prevalent cardiovascular risk factors. In bivariate analyses, TNX levels correlated negatively with age and cardiometabolic risk parameters (blood pressure, glucose) and positively with kidney function; in multivariable regression, TNX was independently associated with age, sex, and height, whereas the associations with cardiometabolic parameters were no longer significant. TNX levels remained unchanged following acute glucocorticoid administration. TNC levels correlated negatively with age and glycated hemoglobin and positively with kidney function in bivariate analyses. These findings support distinct but overlapping roles for TNX and TNC in connective tissue function and extend current knowledge, suggesting both proteins as candidate systemic biomarkers for aging. A further investigation of TNX and TNC in a clinical context could reveal new mechanisms for the detection or prevention of cardiometabolic disease.

## 1. Introduction

Tenascins are a family of extracellular matrix (ECM) glycoproteins involved in developmental processes and connective tissue structure. In addition, studies indicate functions in the modulation of cell adhesion as well as the extracellular regulation of signaling processes [1,2].

The best studied member of the tenascin family is tenascin C (TNC). TNC has been shown to play a role in wound healing, tumorigenesis and inflammatory diseases [3,4]. TNC levels are up-regulated in cardiopulmonary diseases, including dilated cardiomyopathy [5] and atrial fibrillation [6].

Not as much is known about tenascin X (TNX), which is structurally related to TNC [7]. TNX deficiency due to biallelic pathogenic variants of the tenascin XB gene (*TNXB*, MIM *600985) leads to a classic-like form of Ehlers–Danlos syndrome (EDS, MIM #606408), which is clinically characterized by joint hypermobility, easy bruising and vascular fragility and structural cardiac abnormalities (valvulopathies, cardiomyopathy) in some cases [8].

EDS due to *TNXB* variants was discovered serendipitously as a comorbidity in patients with congenital adrenal hyperplasia (CAH) due to 21-hydroxylase deficiency (21-OHD), which is caused by compound heterozygous orhomozygous pathogenic variants in the *CYP21A2* gene that can lead to hyperandrogenemia as well as glucocorticoid and mineralocorticoid deficiency [9]. Both *CYP21A2* and *TNXB* are located adjacent to each other within the highly variable major histocompatibility class III region of chromosome 6, a region which is particularly prone to copy number variation, and hence large deletions in *CYP21A2* sometimes extend into the *TNXB* gene, whose 3′ region on the opposite strand overlaps with *CYP21A2* [10]. Patients with such pathogenic variants in both the *CYP21A2* and *TNXB* genes may be affected by both 21-OHD and EDS, a recently characterized clinical syndrome termed CAH-X [10,11].

In addition, *TNXB* may play a role in atherosclerosis and malignant diseases. TNX was demonstrated to mediate endothelial-to-mesenchymal transition and hence endothelial inflammation and atherosclerosis, via transforming growth factor beta (TGF-β) signaling [12], and TNX expression was shown to be reduced in calcified aortic valves [13]. TNX mRNA and protein expression were down-regulated in commonly occurring cancers including breast and lung cancer, and TNX’s mRNA expression was correlated with survival [14]. Studies on other aspects of TNX function are scarce.

Interestingly, TNX serum levels in patients with 21-OHD were significantly lower than in controls and even in patients without concomitant *TNXB* mutations [15], and conversely, TNX levels in EDS patients were lower than in healthy individuals, even in the absence of *TNXB* mutations in EDS patients [16]. The administration of glucocorticoids has been suggested as a possible explanation of the variation in TNX levels in patients with CAH [15]; however, significant variations in TNX levels have also been observed in other cohorts without *TNXB* mutations or CAH [16]. These data suggest that TNX levels may be related to other not yet identified clinical characteristics that could play a role in the development of cardiovascular or metabolic disease, which is prevalent in patients with EDS or 21-OHD, respectively. Knowledge of the regulatory mechanisms of TNX could lay the foundation for new approaches to the improvement in the diagnosis and management of such diseases.

We therefore explored TNX and TNC levels and their potential associations with clinical and biochemical characteristics in a clinically heterogeneous population of patients including a large proportion with cardiometabolic risk factors. In addition, we investigated the effect of standardized glucocorticoid administration on TNX levels.

## 2. Results

### 2.1. Patient Population and Baseline Characteristics

**Figure 1:**
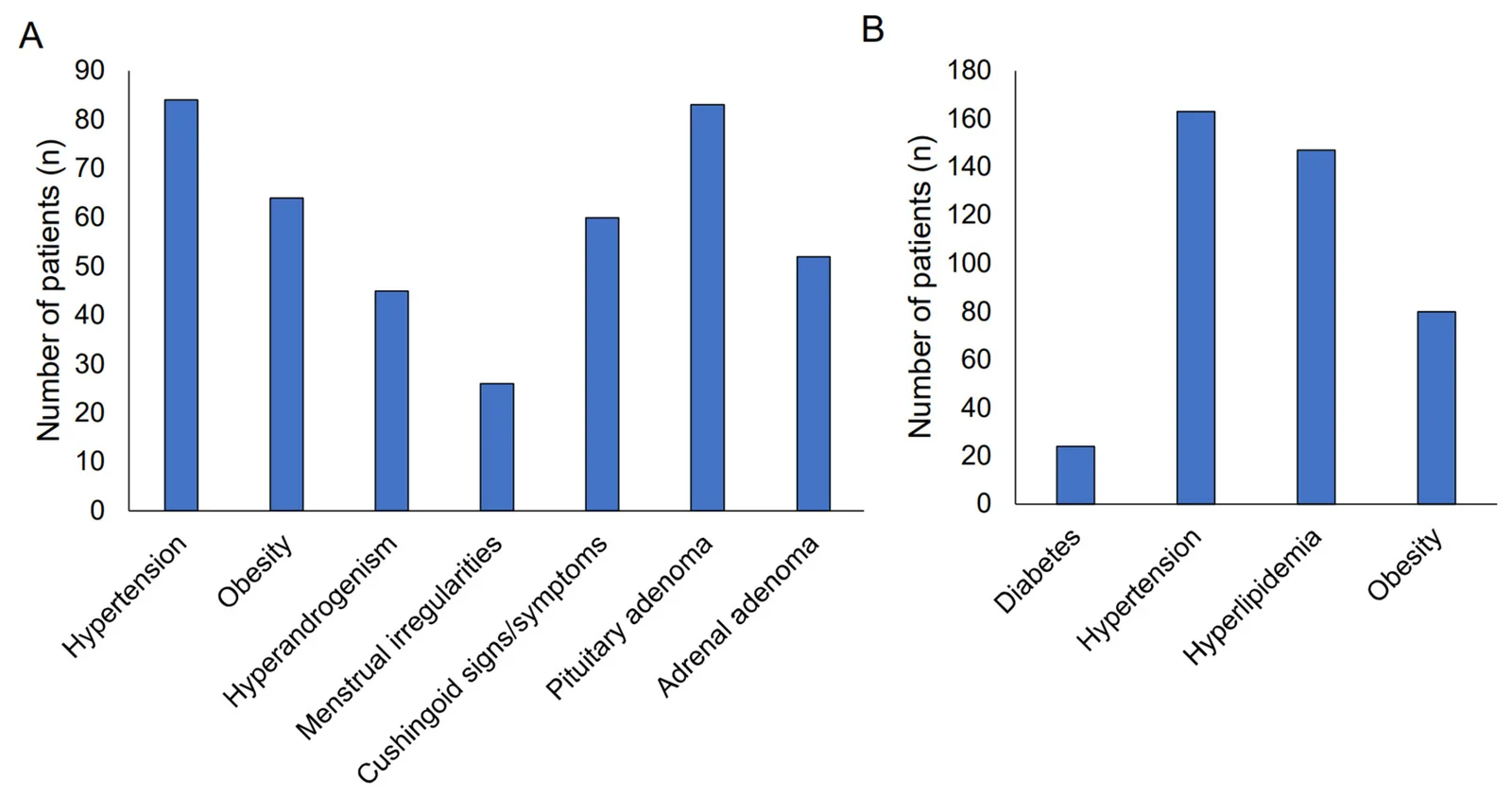
Included patient numbers according to indications for dexamethasone suppression testing (**A**) and prevalence of comorbidities (**B**) in our cohort.

This study included 274 patients (male n = 81; female n = 193) in whom Cushing’s syndrome was excluded by 1 mg overnight dexamethasone suppression testing (DST) in routine endocrine evaluation due to different indications including hypertension and adrenal incidentalomas (Figure 1A). Included patients demonstrated a wide spectrum of cardiovascular risk factors such as hyperlipidemia and obesity (Figure 1B). Baseline biochemical and clinical characteristics are shown in Table 1.

### 2.2. TNX Levels and Clinical Correlates

In the whole cohort, median TNX levels were 190.4 ng/mL (±76.7 ng/mL). We identified two extreme outliers (1227.2 and 1239.0 ng/mL) which were excluded from further calculations. There was a non-significant tendency towards lower levels in men (179.1 ± 73.8 ng/mL) compared to women (193.1 ± 78.9 ng/mL, *p* = 0.060 by Mann–Whitney U test).

**Figure 2:**
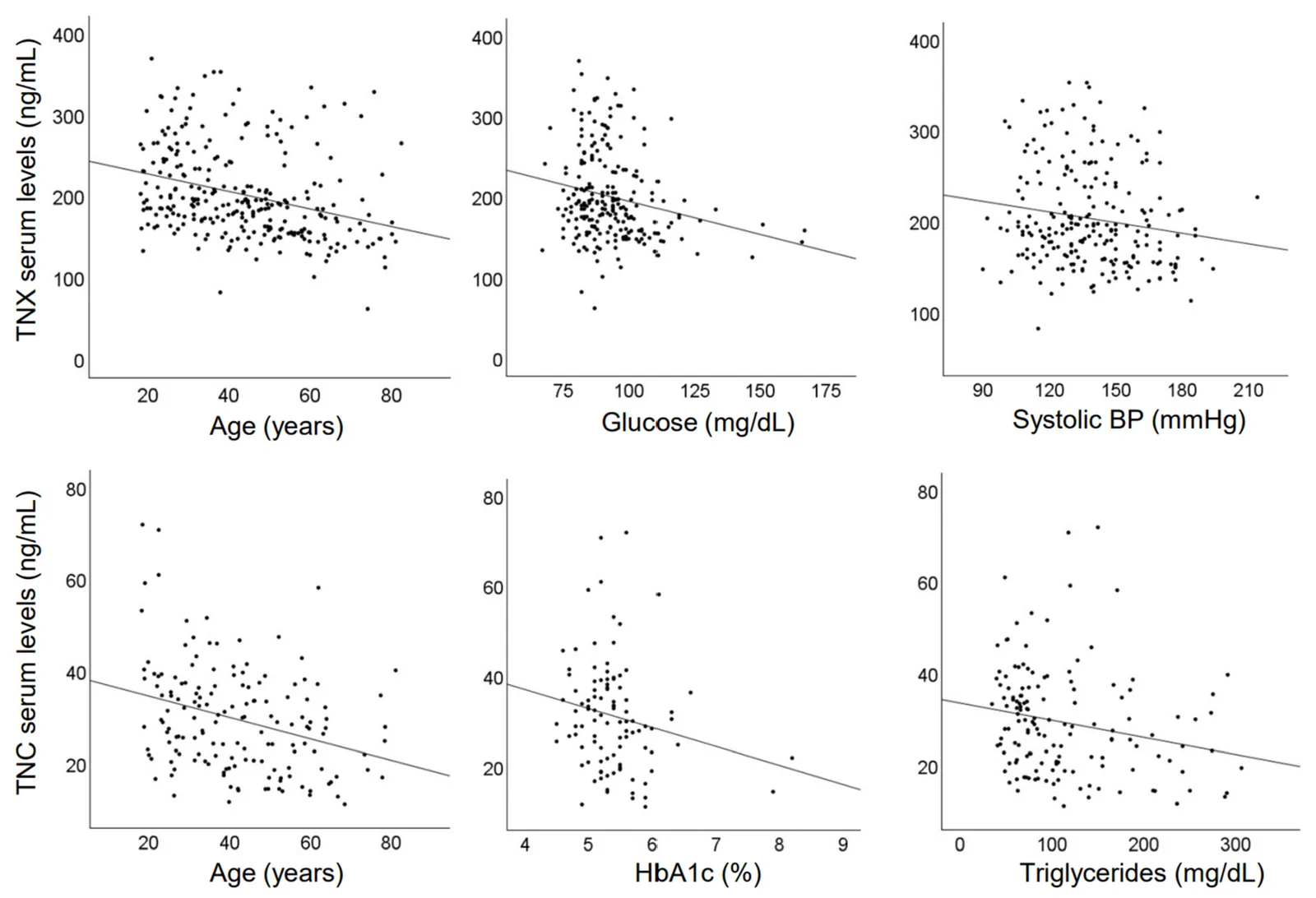
Scatter plots showing correlations between TNX (upper panels) or TNC (lower panels) and cardiometabolic risk parameters. BP—blood pressure; HbA1c—glycated hemoglobin.

TNX levels were significantly negatively correlated with age, blood pressure and fasting glucose (Figure 2) and positively with kidney function and albumin (Table 2).

**Figure 3:**
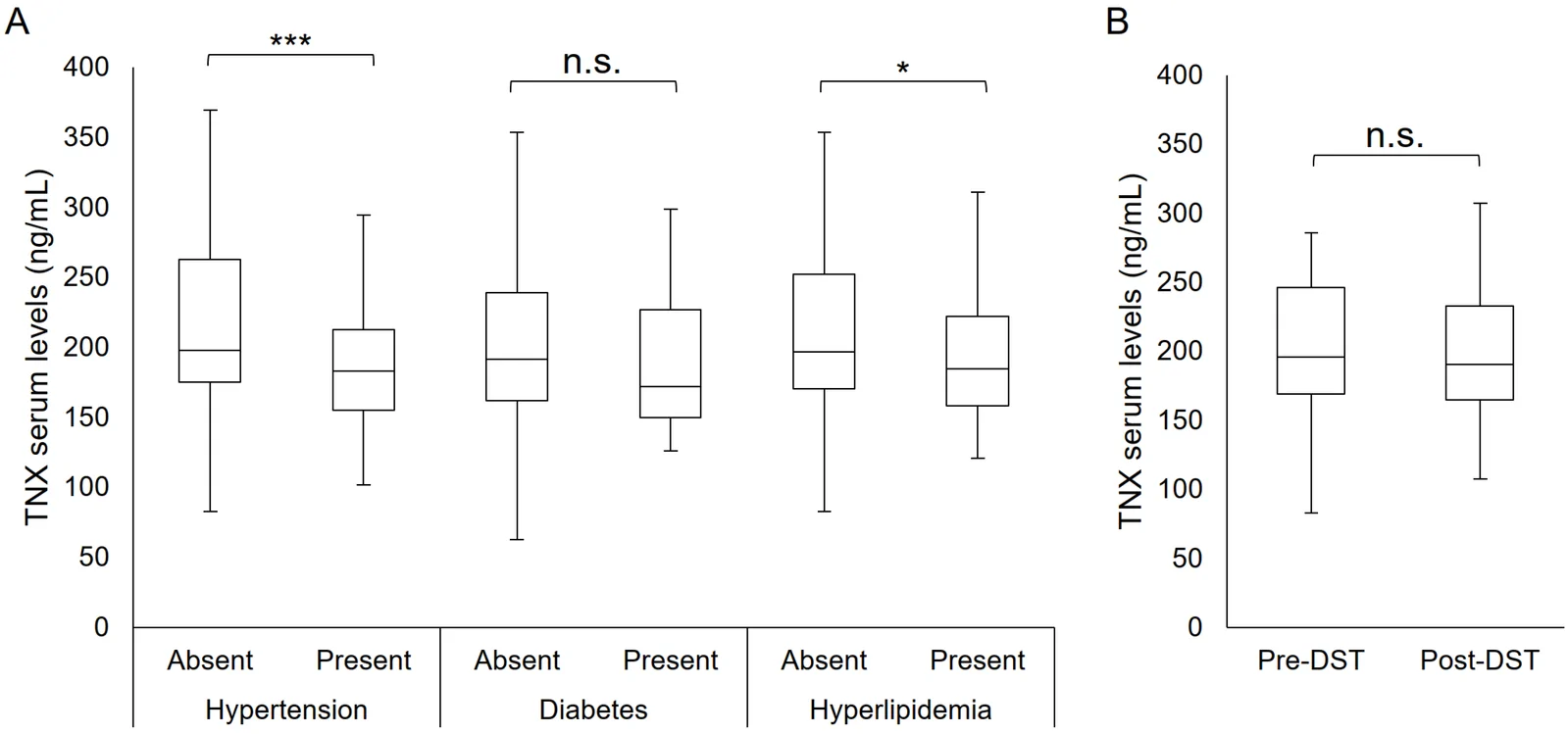
(**A**) Box plots showing TNX levels according to the presence or absence of hypertension, diabetes and hyperlipidemia. (**B**) TNX levels before and after DST. Boxes represent the interquartile range, the middle denotes the median and whiskers are the minimum/maximum. Differences between groups were tested by Mann–Whitney U tests (**A**) or a paired samples Wilcoxon test (**B**). DST—dexamethasone suppression test; n.s.—not significant. * *p* < 0.05, *** *p* < 0.001

To account for patients on antidiabetic, antihypertensive or lipid-lowering medication, we compared TNX levels between patients with and without diabetes, hypertension or hyperlipidemia, demonstrating significantly lower levels of TNX in patients with hypertension or hyperlipidemia (Table 3 and Figure 3A).

In addition, in male patients, TNX levels were significantly correlated with creatine kinase (CK) and alanine aminotransferase (ALT). In female patients, we detected a significant positive correlation of TNX with height and negative correlations with HbA1c, triglycerides and BMI (Table 2).

Clinically relevant and non-collinear parameters were included in multivariable regression analysis, which identified age, sex and height as significantly associated with TNX levels (Table 2). Specifically, for every 1-year increase in age, TNX levels decreased by approximately 0.4%, holding all other variables constant. For every 1 cm increase in height, TNX levels increased by approximately 0.9%, holding all other variables constant. Male patients had significantly lower TNX levels than female patients (β = −0.131, 95%-CI: −0.242;−0.020, *p* < 0.05). No violations of modeling assumptions were observed.

No correlation was detected between TNX levels and inflammatory parameters (C-reactive protein (CRP) or leukocyte count), whilst inflammatory parameters were significantly correlated with cardiovascular risk parameters: CRP-BMI (ρ = 0.598, *p* < 0.001), CRP-blood pressure (systolic ρ = 0.147, *p* = 0.032, diastolic ρ = 0.172, *p* = 0.012).Within endocrine parameters, TNX was strongly associated with prolactin levels in women. After the exclusion of patients on cabergoline (n = 12) and patients with prolactin levels > 50 ng/mL (n = 8), there was still a significant correlation between TNX and prolactin levels in women (ρ = 0.243, *p* = 0.029). Since there was a significant association between age and prolactin levels in this subgroup (ρ = −0.223, *p* = 0.044 in Spearman Rank correlation), we suggest that the association between TNX and prolactin is at least partially age-dependent.

### 2.3. Influence of Glucocorticoid Administration on TNX Levels

To determine any influence of acute dexamethasone administration on TNX levels, we measured TNX after DST in a subgroup of individuals (n = 37). We detected no significant difference in TNX levels before (mean 206.3 ± 58.6 ng/mL) and after dexamethasone administration (median 190.4 ± 67.8 ng/mL, *p* = 0.667 in paired samples Wilcoxon test) (Figure 3B). Similarly, no significant differences between TNX levels before and after DST were detected when men and women were analyzed separately.

### 2.4. TNC Levels and Clinical Correlates

TNC levels were determined in a subgroup of individuals included in this study (n = 154; male n = 47; female n = 107). Median TNC levels were 28.4 ng/mL (±16.2 ng/mL), without a significant difference between men (27.8 ± 15.1 ng/mL) and women (29.1 ± 16.3 ng/mL, *p* = 0.914 by Mann–Whitney U test). Similarly to TNX, TNC levels were negatively associated with age and positively with kidney function (Table 4). In addition, TNC levels were significantly positively correlated with alkaline phosphatase levels and with IGF-1 levels.

Interestingly, we observed a significant negative relationship of HbA1c (but not fasting glucose) and triglycerides (but not cholesterol) with TNC levels (Figure 2). These correlations remained significant after excluding patients on lipid-lowering medication (TNC-triglycerides ρ = −0.292, *p* = 0.001) or antidiabetic medication (TNC-HbA1c ρ = −0.210, *p* = 0.046), respectively, from analysis.

Multivariable regression analysis observed no significant associations with TNC levels in our cohort (Table 4).

No variations in TNC levels were detected in patients with diabetes, hyperlipidemia or hypertension compared to those without (all *p* > 0.05 in Mann–Whitney U tests); moreover, no correlation between TNC levels and inflammatory parameters (CRP, leukocyte count) was observed.

## 3. Discussion

We show here the clinical correlates of serum TNX and TNC levels in a heterogenous cohort of individuals with prevalent cardiometabolic risk factors. We identified age, sex and height as independently associated with TNX levels, as well as bivariate correlations of TNX with creatine kinase, albumin, cardiometabolic risk parameters (glucose, blood pressure and triglycerides) and prolactin levels. We did not detect any acute effect of dexamethasone administration on TNX levels. TNC levels correlated with age, kidney function, glucose metabolism and IGF-1 levels in bivariate analyses only.

No previous data are available on TNX levels and their clinical correlates in larger cohorts. Consequently, no normal range in control or patient cohorts has been established. Previous studies in smaller or clinically less broadly characterized cohorts have used different detection methods, impeding comparability [15,17]. Our observation of the correlation between TNX and CK levels in men was previously undocumented. CK elevations are typically associated with skeletal muscle damage or myopathies, and hence this correlation provides evidence for the best documented action of TNX in connective tissue function. Corroborating this association, height in women and IGF-1 levels in the whole cohort were also significantly correlated with TNX levels. As the main mediator of growth hormone function, IGF-1 acts in the stimulation of the anabolic effects of muscle and connective tissue [18].

The positive correlations of TNX with albumin and kidney function may be secondary to an age-related decline in all three parameters; alternatively, a fraction of serum TNX may be bound by albumin.

In bivariate analyses, we observed an association between lower TNX levels and several cardiometabolic risk parameters including fasting glucose, systolic blood pressure and triglyceride levels, which was no longer significant in multivariable analyses. While these relationships could potentially be secondary to aging, cardiometabolic risk and atherosclerosis are linked to inflammation via endothelial dysfunction and vascular remodeling [19], and TNX was shown to mediate TGF-β function by regulating its bioavailability and activation [20]. Lower TNX levels could therefore also indicate a loss of compensatory mechanisms normally promoting tissue stability.

Even though tissue-specific TNX expression is known to play a role in organogenesis and varies in different embryonic developmental stages [21], no previous data exist showing a correlation between circulating TNX levels and adult age. The positive associations of TNX with IGF-1 and prolactin may also be age-dependent since IGF-1 levels decline continuously in adult life [22], and prolactin decreases with age in women [23]. Alternatively, the association of TNX with prolactin may reflect the less well-known roles of prolactin in cartilage survival, such as bone turnover and immune modulation [24,25,26,27,28,29].

It has been suggested that ongoing glucocorticoid therapy in CAH may offer an explanation for the observation that TNX serum levels in patients with 21-OHD were significantly lower than in controls and even in patients without concomitant *TNXB* mutations [15]. We observed that a single dose of 1 mg dexamethasone does not acutely influence TNX levels approximately 9 h later. Notably, this acute exposure differs substantially from chronic glucocorticoid therapy in patients with CAH, and our data therefore do not exclude the effect of long-term glucocorticoid treatment on TNX levels. We suggest that the significant association of TNX with body height could contribute to the lower levels in 21-OHD since patients with 21-OHD are well known to often have reduced adult stature [30]. Furthermore, the absence of a significant effect should be interpreted with caution given the small size of this subgroup. Further studies are necessary to explore the effect of chronic glucocorticoid treatment, other glucocorticoids or different modes of administration in a larger cohort.

The clinical associations we observed for TNC show overlaps as well as divergences with the TNX correlates. Both parameters correlate with age, cardiometabolic parameters and kidney function in bivariate analyses. TNC was more closely related to glycated hemoglobin (HbA1c) rather than fasting glucose, as well as triglycerides. There is considerable heterogeneity in the previous literature connecting TNC levels and age as well as cardiometabolic risk parameters [31,32,33]. Caution is warranted with the interpretation of these discrepancies. Studied patient cohorts were very diverse, and compensatory mechanisms may govern the direction of some observed relationships. Indeed, TNC was demonstrated to exert pro- and anti-inflammatory function in different contexts [21]. The relationships of TNC with alkaline phosphatase and IGF-1 are consistent with the known functions of TNC in bone and cartilage [34] and with its binding of insulin-like growth factor-binding proteins [35]. We did not detect significant correlations between TNC and systemic inflammatory parameters (CRP or leukocyte count), which is in agreement with some previous studies [36] but not others [33,37].

This study has several limitations that should be discussed. Our retrospectively identified correlations and regression do not allow for any inference of causality; prospective studies are necessary to confirm our observations. We measured TNX and TNC serum levels by immunoassay, a method generally susceptible to matrix interference; furthermore, the preferential detection of specific TNX isoforms by the assay cannot be excluded. Although we have no evidence suggesting that matrix interference is a significant confounder in our study, mass spectrometry-based methods would be more sensitive for such measurements. However, due to the size of our cohort and the homogeneity of sample handling, we hope to minimize other systematic bias. Furthermore, immunoassay-based methods currently remain more easily accessible in most clinical contexts. Finally, the serum levels of both TNX and TNC may diverge from tissue-specific expression and regulation; hence, lower serum levels may not preclude tissue-specific up-regulation and relevant localized functions.

In summary, we identified overlapping but distinct clinical correlates of TNX and TNC serum levels in a single-center cohort of patients with prevalent cardiometabolic risk factors. These findings have several implications. First, age, sex and height should be taken into account when TNX levels are compared between patient groups and when reference ranges are established. This is particularly relevant for 21-OHD and EDS, in which lower TNX levels have been reported [15,16] and which may differ from control populations in terms of age, sex distribution or stature [30]. Second, our data indicate that TNX measurements are not acutely altered by a single dose of dexamethasone, whereas the effect of chronic glucocorticoid treatment remains to be determined. Third, the inverse association with age suggests that circulating TNX may reflect age-related changes in connective tissue, which should be addressed in longitudinal studies. In contrast, TNC was not independently associated with any clinical parameter, suggesting that in the absence of acute tissue injury, circulating TNC varies only modestly with clinical characteristics. A further functional exploration of the tenascins in different clinical contexts could uncover new mechanisms for the detection or prevention of cardiometabolic diseases.

## 4. Methods and Materials

### 4.1. Study Design and Patient Population

This study is a retrospective cohort analysis that was approved by the institutional review board of the Medical University of Vienna (EK 1336/2021) and was conducted in accordance with the Declaration of Helsinki.

This study included 274 consecutive patients who were evaluated at the endocrine outpatient clinic of our department between 2020 and 2022 for different reasons including adrenal adenoma, obesity, hypertension and hirsutism (cf. Results Section). Dexamethasone suppression testing (DST) was performed in all patients by the oral intake of 1 mg dexamethasone at 11pm, followed by a fasted 8 am morning blood draw to determine the suppression of endogenous cortisol levels. This study includes only patients in whom Cushing’s syndrome could be subsequently ruled out as demonstrated by the adequate suppression of post-dexamethasone cortisol levels ≤ 1.8 mcg/dl and additional diagnostic testing as clinically indicated in some patients. Other inclusion criteria were sufficient data availability and an age of 18 years or older. Patients on oral contraceptive treatment or other glucocorticoid medication (oral, topical, inhaled or injected) and those with a past history of Cushing’s syndrome were excluded.

### 4.2. Data Collection

Data on medical history and clinical and biochemical parameters, including age, gender, height, weight, blood pressure, blood count and clinical chemistry, endocrine parameters, concomitant medication and comorbidities, were extracted from hospital records at the time when dexamethasone suppression testing was performed (usually the time of first presentation at our clinic).

### 4.3. Parameters and Assays

Venous blood samples were drawn during routine visits at our outpatient clinic. Standard chemistry, blood count and endocrine parameters were measured before dexamethasone suppression testing by our institution’s certified laboratory using routine methods (www.kimcl.at, accessed on 16 October 2026). Prolactin levels were measured by electrochemiluminescence immunoassays (ECLIA; Roche, Cobas), and IGF-1 (insulin-like growth factor 1) levels were measured by chemoluminescence immunoassays (DiaSorin, Liaison).

TNX and TNC levels were measured in serum samples that had been centrifuged and frozen immediately after blood draw at −20 °C in the course of dexamethasone suppression testing. For correlation and regression analyses, only the samples taken before dexamethasone intake were used. Samples taken after dexamethasone intake were only used for the analyses comparing TNX levels before and after dexamethasone. TNX levels were quantified by a sandwich enzyme-linked immunosorbent assay (ELISA) kit from RayBiotech (Peachtree Corners, GA, USA, Human Tenascin-X ELISA Kit, ELH-TNSNX, sensitivity 0.052 ng/mL, detection range 0.052–12.5 ng/mL). TNC levels were quantified by sandwich ELISA from Abcam (Cambridge, UK, Human Tenascin-C ELISA kit, ab213831, sensitivity < 10 pg/mL, detection range 93.7–6000 pg/mL). The kit detects natural and recombinant human TNC; the recombinant standard spans amino acids G23–P625, a region common to all known TNC splice variants.

The glomerular filtration rate (GFR) was estimated using the CKD-EPI 2021 refit equation [38].

Body mass index (BMI) was calculated as weight (kg)/height(m)^2^, and obesity was defined as a BMI ≥ 30 kg/m^2^. The presence of diabetes mellitus was defined as fasting glucose ≥ 126 mg/dL, glycated hemoglobin (HbA1c) ≥ 6.5% or current antidiabetic therapy [39]. The presence of hyperlipidemia was defined as total cholesterol ≥ 200 mg/dL or triglycerides ≥ 150 mg/dL or current lipid-lowering therapy [40].

### 4.4. Statistical Analysis

The normality of distribution in quantitative variables was analyzed by Shapiro–Wilk tests. Data are shown as means (± S.D., standard deviation) for normally distributed data and as medians (±IQR, interquartile range) for non-normally distributed data. Differences between groups were assessed with independent samples Student’s *t*-tests or Mann–Whitney U tests, as appropriate. Paired analysis (pre- and post-DST) was computed with a paired samples Wilcoxon test without a control group. Associations between variables were analyzed with Spearman Rank correlation analyses. Bivariate relationships were analyzed on pairwise complete cases.

For multivariable regression analysis, missing values were handled using Multiple Imputation by Chained Equations (MICE). This approach was selected under the assumption that data are Missing At Random (MAR) and to utilize all available observations. The imputation model incorporated all variables from the two regression analyses alongside additional auxiliary variables to enhance predictive accuracy. A correlation-based predictor matrix was generated. Fifty imputed datasets were generated, each undergoing 20 iterations. Final parameter estimates and standard errors were pooled across the imputed datasets using Rubin’s rules. For the outcome TNC, only patients with non-missing values in the original dataset were analyzed with the imputed covariables (n = 154).

TNX and TNC were log-transformed to satisfy the linear regression assumptions of normally distributed residuals. Univariable linear regressions were computed for each variable, followed by the multivariable adjusted linear regression. For interpretation, the results from the log-transformed models were back-transformed and interpreted as percentage differences. Percentage changes from the coefficients were approximated as β * 100%, which closely agrees with the exact transformation (e^β^ − 1) * 100% for coefficients of small magnitude. Regression analyses were repeated on the complete cases for sensitivity analysis.

Two-sided *p*-values < 0.05 were considered statistically significant. Analyses were performed by SPSS Statistics Version 29 (IBM) and by R version 4.6.1.

## Figures and Tables

**Table 1:** Baseline parameters.

	All	Male	Female	*p*	Reference Range
N (%)	274	81 (29.6)	193 (70.4)		
Age (years)	43.2 (25.2)	43.7 (27.0)	41.7 (23.6)	n.s.	
Height (cm)	168.0 (11.0)	177.8 (7.4)	165.6 (6.2)	<0.001	
Weight (kg)	78.0 (27.0)	90.0 (22.0)	72.0 (28.0)	<0.001	
BMI (kg/m^2^)	26.7 (8.6)	28.1 (6.4)	26.2 (9.9)	0.03	
Systolic BP (mmHg)	137.0 (31.0)	146.0 (17.0)	131.0 (31.5)	<0.001	<120
Diastolic BP (mmHg)	86.0 (18.8)	89.0 (12.4)	85.0 (18.8)	n.s.	<70
Creatinine (mg/dL)	0.73 (0.2)	0.86 (0.19)	0.70 (0.16)	<0.001	0.7–1.2 (M), 0.5–0.9 (F)
Blood urea nitrogen (mg/dL)	12.2 (4.8)	12.8 (4.9)	11.9 (4.9)	0.02	6–20
eGFR (ml/min/1.73 m^2^)	106.4 (24.0)	103.9 (18.4)	106.6 (23.5)	n.s.	>55
Uric acid (mg/dL)	4.6 (1.7)	5.5 (1.8)	4.3 (1.3)	<0.001	3.4–7.0 (M), 2.4–5.7 (F)
Albumin (g/L)	46.4 (3.1)	46.8 (3.1)	46.2 (3.0)	n.s.	35–52
Alkaline phosphatase (U/L)	67.0 (25.0)	71.0 (23.0)	66.0 (26.3)	n.s.	40–130 (M), 35–105 (F)
AST (U/L)	21.0 (8.0)	23.0 (11.5)	20.0 (7.0)	<0.001	<50 (M), <35 (F)
ALT (U/L)	22.0 (15.0)	29.0 (23.5)	21.0 (12.3)	<0.001	<50 (M), <35 (F)
GGT (U/L)	19.0 (17.0)	28.0 (22.5)	17.0 (10.0)	<0.001	<60 (M), <40 (F)
CK (U/L)	88.0 (68.5)	85.0 (50.3)	109.5 (91.5)	<0.001	<190 (M), <170 (F)
Glucose (mg/dL)	91.0 (15.0)	95.0 (15.0)	88.0 (13.0)	<0.001	74–109
HbA1c (%)	5.4 (0.6)	5.4 (0.4)	5.4 (0.6)	n.s.	4.0–6.0
Triglycerides (mg/dL)	94.0 (70.0)	107.0 (78.0)	85.0 (59.5)	0.004	<150
Cholesterol (mg/dL)	187.0 (58.0)	185.5 (53.2)	189.0 (55.5)	n.s.	<200
HDL (mg/dL)	57.0 (21.0)	48.0 (19.5)	60.0 (23.0)	<0.001	>55 (M), >65 (F)
LDL (mg/dL)	106.9 (44.4)	109.6 (48.7)	106.8 (44.0)	n.s.	<116
CRP (mg/dL)	0.13 (0.28)	0.14 (0.25)	0.12 (0.31)	n.s.	<0.5
Leukocyte count (G/L)	6.4 (2.6)	6.5 (2.0)	6.4 (2.9)	n.s.	3.64–8.46 (M), 3.8–9.39 (F)
TSH (µIU/mL)	1.7 (1.2)	1.7 (1.0)	1.6 (1.2)	n.s.	0.27–4.2
fT4 (ng/dL)	1.2 (0.3)	1.3 (0.3)	1.2 (0.3)	n.s.	0.76–1.66
IGF-1	196.0 (88.8)	204.0 (89.0)	204.6 (64.8)	n.s.	*
Prolactin	11.8 (12.8)	8.8 (10.2)	13.2 (14.6)	0.02	4.0–15.2 (M), 4.8–23.3 (F)

Shown are means/medians (S.D./IQR). Differences between male and female groups were tested with independent samples *t*-tests or Mann–Whitney U tests. Assay-specific reference ranges are provided. * Age- and sex-dependent normal ranges. ALT—alanine aminotransferase; AST—aspartate aminotransferase; BMI—body mass index; BP—blood pressure; CK—creatine kinase; CRP—C-reactive protein; eGFR—estimated glomerular filtration rate; F—females; GGT—gamma glutamyl transferase; HbA1c—glycated hemoglobin; HDL—high-density lipoprotein; IGF-1—insulin-like growth factor 1; LDL—low-density lipoprotein; M—males. n.s.—not significant.

**Table 2:** Correlations between TNX serum levels and other clinical and biochemical parameters.

	Bivariate Correlation						Multivariable Regression		
	All		Male		Female		All		
	R	*p*	R	*p*	R	*p*	Estimate (Lower CI; Upper CI)	Std. Err.	*p*
Sex (f:m)							−0.1312 (−0.2420; −0.0203)	0.0563	0.021
Age	−0.37	<0.001	−0.37	0.001	−0.37	<0.001	−0.0038 (−0.0075; −0.0001)	0.0019	0.042
Height	0.11	n.s.	0.14	n.s.	0.27	<0.001	0.0093 (0.0036; 0.0151)	0.0029	0.002
Weight	−0.01	n.s.	0.21	n.s.	−0.02	n.s.			
BMI	−0.10	n.s.	0.22	n.s.	−0.18	0.02			
Systolic BP	−0.17	0.01	−0.11	n.s.	−0.14	n.s.	−0.0009 (−0.0027; 0.0010)	0.0010	0.367
Diastolic BP	−0.12	n.s.	−0.17	n.s.	−0.09	n.s.			
eGFR	0.22	<0.001	0.27	0.020	0.20	0.006	0.0002 (−0.0026; 0.0031)	0.0014	0.871
Blood urea nitrogen	−0.19	0.002	−0.17	n.s.	−0.17	0.02			
Uric acid	−0.08	n.s.	−0.05	n.s.	−0.04	n.s.			
Albumin	0.17	0.006	0.15	n.s.	0.20	0.007	0.0077 (−0.0052; 0.0206)	0.0066	0.239
Alkaline phosphatase	0.02	n.s.	0.13	n.s.	0.00	n.s.			
AST	0.11	n.s.	0.18	n.s.	0.12	n.s.			
ALT	0.049	n.s.	0.33	0.006	0.00	n.s.	0.0014 (−0.0008; 0.0036)	0.0011	0.204
GGT	−0.10	n.s.	0.00	n.s.	−0.08	n.s.			
CK	0.11	n.s.	0.41	0.002	0.04	n.s.	0.0002 (−0.0003; 0.0006)	0.0002	0.440
Glucose	−0.22	0.001	−0.16	n.s.	−0.25	0.001	0.000 (−0.0030; 0.0031)	0.0015	0.978
HbA1c	−0.15	n.s.	−0.01	n.s.	−0.19	0.05			
Triglycerides	−0.12	n.s.	0.02	n.s.	−0.16	0.03			
Cholesterol	−0.06	n.s.	0.01	n.s.	−0.10	n.s.			
HDL	0.11	n.s.	−0.01	n.s.	0.10	n.s.			
LDL	−0.06	n.s.	0.06	n.s.	−0.11	n.s.			
CRP	−0.10	n.s.	−0.10	n.s.	−0.09	n.s.			
Leukocyte count	−0.01	n.s.	−0.04	n.s.	0.01	n.s.			
TSH	0.04	n.s.	0.10	n.s.	0.03	n.s.			
fT4	−0.06	n.s.	−0.05	n.s.	−0.04	n.s.			
IGF-1	0.18	0.03	0.11	n.s.	0.20	n.s.			
Prolactin *	0.24	0.008	0.10	n.s.	0.24	0.03			

Table showing bivariate Spearman’s correlation coefficients on pairwise complete cases and multivariable regression analysis. * Excluding patients on cabergoline or with disorders of prolactin secretion. ALT—alanine aminotransferase; AST—aspartate aminotransferase; BMI—body mass index; BP—blood pressure; CI—confidence interval; CK—creatine kinase; CRP—C-reactive protein; eGFR—estimated glomerular filtration rate; f—female; fT4—free thyroxine; GGT—gamma glutamyl transferase; HbA1c—glycated hemoglobin; HDL—high-density lipoprotein; IGF-1—insulin-like growth factor 1; LDL—low-density lipoprotein; m—male; n.s.—not significant; Std. Err.—standard error.

**Table 3:** TNX serum levels according to presence or absence of hypertension, diabetes and hyperlipidemia.

	Absent	Present	N Affected (%)	*p*
Hypertension	197.8 (87.6)	183.2 (57.2)	160 (59.5)	<0.001
Diabetes	191.4 (77.1)	172.0 (76.9)	24 (8.9)	0.095
Hyperlipidemia	197.0 (81.8)	184.5 (64.0)	146 (54.3)	0.020

Table showing medians (IQR) of TNX serum levels (ng/mL) according to presence or absence of comorbidities. Differences between groups were tested with Mann–Whitney U tests.

**Table 4:** Correlations between TNC serum levels and other clinical and biochemical parameters.

	Bivariate Correlation						Multivariable Regression		
	All		Male		Female		All		
	R	*p*	R	*p*	R	*p*	Estimate (Lower CI; Upper CI)	Std. Err.	*p*
Sex (m:f)							−0.0008 (−0.2207; 0.2192)	0.1096	0.994
Age	−0.31	<0.001	−0.25	n.s.	−0.34	<0.001	−0.0037 (−0.0132; 0.0057)	0.0047	0.432
Height	0.04	n.s.	−0.25	n.s.	0.12	n.s.			
Weight	−0.11	n.s.	−0.21	n.s.	−0.07	n.s.			
BMI	−0.13	n.s.	−0.08	n.s.	−0.13	n.s.			
Systolic BP	0.02	n.s.	0.07	n.s.	0.00	n.s.			
Diastolic BP	0.02	n.s.	−0.02	n.s.	0.02	n.s.			
eGFR	0.21	0.009	0.21	n.s.	0.22	0.03	0.0003 (−0.0076; 0.0081)	0.0039	0.947
Blood urea nitrogen	−0.20	0.01	0.03	n.s.	−0.27	0.005			
Uric acid	−0.15	n.s.	−0.21	n.s.	−0.15	n.s.			
Albumin	0.02	n.s.	−0.01	n.s.	0.07	n.s.			
Alkaline phosphatase	0.17	0.04	0.27	n.s.	0.11	n.s.	0.0040 (−0.0007; 0.0088)	0.0024	0.096
AST	−0.02	n.s.	−0.10	n.s.	0.01	n.s.			
ALT	−0.05	n.s.	−0.01	n.s.	−0.10	n.s.			
GGT	−0.15	n.s.	−0.23	n.s.	−0.17	n.s.			
CK	0.05	n.s.	0.05	n.s.	0.04	n.s.			
Glucose	−0.04	n.s.	−0.06	n.s.	−0.05	n.s.			
HbA1c	−0.22	0.03	−0.14	n.s.	−0.28	0.02	0.0178 (−0.1883; 0.2240)	0.1027	0.863
Triglycerides	−0.27	0.001	−0.24	n.s.	−0.31	0.002	−0.0016 (−0.0032; 0.0001)	0.0008	0.059
Cholesterol	−0.06	n.s.	−0.20	n.s.	0	n.s.			
HDL	0.08	n.s.	−0.05	n.s.	0.12	n.s.			
LDL	0.01	n.s.	−0.17	n.s.	0.08	n.s.			
CRP	−0.07	n.s.	−0.13	n.s.	−0.04	n.s.			
Leukocyte count	0.16	n.s.	0.17	n.s.	0.16	n.s.			
TSH	0.09	n.s.	−0.04	n.s.	0.14	n.s.			
fT4	0.10	n.s.	0.07	n.s.	0.10	n.s.			
IGF-1	0.43	<0.001	0.39	0.03	0.44	0.001	0.0012 (−0.0003; 0.0027)	0.0007	0.113
Prolactin *	0.10	n.s.	−0.08	n.s.	0.14	n.s.			

Table showing bivariate Spearman’s correlation coefficients on pairwise complete cases and multivariable regression analysis. * Excluding patients on cabergoline or with disorders of prolactin secretion. ALT—alanine aminotransferase; AST—aspartate aminotransferase; BMI—body mass index; BP—blood pressure; BUN—blood urea nitrogen; CI—confidence interval; CK—creatine kinase; CRP—C-reactive protein; eGFR—estimated glomerular filtration rate; f—female; fT4—free thyroxine; GGT—gamma glutamyl transferase; HbA1c—glycated hemoglobin; HDL—high-density lipoprotein; IGF-1—insulin-like growth factor 1; LDL—low-density lipoprotein; m—male; n.s.—not significant; Std. Err.—standard error.

## Data Availability

The data supporting the findings of this study are available from the corresponding author upon reasonable request. Some data are not publicly available due to privacy or ethical restrictions.
